# ﻿Species richness and endemism patterns of Sternorrhyncha (Insecta, Hemiptera) in China

**DOI:** 10.3897/zookeys.1178.107007

**Published:** 2023-09-07

**Authors:** Zhengxue Zhao, Xueli Feng, Yubo Zhang, Yingjian Wang, Zhengxiang Zhou, Tianlei Liu

**Affiliations:** 1 College of Agriculture, Anshun University, Anshun, China Anshun University Anshun China

**Keywords:** Biogeography, ecology environmental variables, normalized difference vegetation index, phytophagous insect, species richness

## Abstract

One of the main goals in biogeography and ecology is the study of patterns of species diversity and the driving factors in these patterns. However, such studies have not focused on Sternorrhyncha in China, although this region hosts massive species distribution data. Here, based on the 15,450 distribution records of Sternorrhyncha species in China, we analyzed patterns in species richness and endemism at 1° × 1° grid size and determined the effects of environmental variables on these patterns using correlations analysis and the model averaging approach. We found that species richness and endemism of Sternorrhyncha species are unevenly distributed, with high values in the eastern and southeastern coastal regions of mainland China, as well as Taiwan Island. Furthermore, the key factors driving species richness and endemism patterns are inconsistent. Species richness patterns were strongly affected by the normalized difference vegetation index, which is closely related to the feeding habits of Sternorrhyncha, whereas endemism patterns were strongly affected by the elevation range. Therefore, our results indicate that the range size of species should be considered to understand the determinants of species diversity patterns.

## ﻿Introduction

Macro-scale patterns of species richness are central to many fundamental questions in ecology and biogeography (MacArthur 1972; Huston 1994; Rosenzweig 1995). Species richness is unevenly distributed across a region (Gaston 2000), which means that some areas within a region have high species richness while others have low species richness. Exploring these patterns has fascinated ecologists and biogeographers for decades (Lobo et al. 2002; Qian et al. 2009a; Feng et al. 2016) and it is critical for understanding species evolution and developing conservation strategies (Pennisi 2005; Zhang et al. 2015; Zhao et al. 2016). While previous studies have found that energy availability is the most important factor in determining species richness patterns (Currie 1991; Hawkins et al. 2003), subsequent studies have reported other factors as more important (Araújo et al. 2008; Li et al. 2019; Lyu et al. 2020). Therefore, there is currently disagreement about the relative contributions of environmental factors, but it is believed that multiple environmental factors interact to influence the patterns of species richness (Wang et al. 2012). Interestingly, several studies have found that the impact of environmental variables on species diversity patterns depends on the range size of species (Svenning and Skov 2007; Liu et al. 2017). Specifically, richness patterns of species are strongly affected by modern environmental factors, whereas those of range-restricted species (also known as endemic species) are strongly affected by historical climate change or topographic heterogeneity (Schuldt and Assmann 2009; Liu et al. 2017; Sosa and Loera 2017). Therefore, we must consider the range size of species to fully understand the causes and mechanisms of patterns of species richness (Schuldt and Assmann 2009).

In comparison to vertebrates and plants, relatively few studies have been conducted on the species richness pattern of insects, despite the fact that insects have the most species (Diniz-Filho et al. 2010; Economo et al. 2018). Thus, the key to revealing the underlying mechanism of global biodiversity is to strengthen the research on the formation mechanism of insect diversity patterns. The Sternorrhyncha suborder, a tiny sucking phytophagous insect of Hemiptera, consists of approximately 18,700 species worldwide (Drohojowska et al. 2020). This suborder comprises four groups: aphids, scale insects, whiteflies and psyllids. The Sternorrhyncha is highly morphologically diverse and widely thought to be of a monophyletic lineage (Drohojowska et al. 2020). Many Sternorrhyncha insects, such as *Myzuspersicae* (Sulzer, 1776), *Dysmicoccusbrevipes* (Cockerell, 1893) and *Phenacoccusmadeirensis* Green, 1923, are major agricultural pests and invasive species. However, some species are beneficial to humans in various ways. Scale insects, for example, provide many useful materials such as red dyes, waxes, resins and medicines (Kondo et al. 2018). Furthermore, many Sternorrhyncha species have become biological indicators of zoogeographical regions and model taxa (e.g., aphids) for biogeography research (Wang et al. 2017). To date, studies on Sternorrhyncha have primarily focused on taxonomy and the prediction of potential distribution areas of some invasive species under climate change (Wei et al. 2019, 2020), but macro-scale patterns of species diversity and the underlying driving factors of these patterns have not been thoroughly investigated, particularly in China, which has the one of largest number of Sternorrhyncha species (Liu et al. 2009; Li 2011; Wei et al. 2016; Yan and Bai 2017).

Some researchers have investigated the species diversity patterns of all Sternorrhyncha in specific regions of China, or some taxa of Sternorrhyncha throughout China. Liu et al. (2009), for example, investigated the distributional patterns of aphid diversity in China using the inverse distance-weighted method. This study, however, did not quantify the effects of environmental factors on species distribution patterns. Wei et al. (2016) discovered that current climate is a major determinant of scale insect diversity patterns in China. Li et al. (2019) investigated the species richness patterns of Hemipteran insects and their relationships with environmental factors in the Qinghai-Tibetan Plateau, identifying climate change since the Last Glacial Maximum and habitat heterogeneity as the main driving factors for Sternorrhyncha. As previously stated, while some research has been conducted in China on the causes of Sternorrhyncha richness patterns, it is still insufficient to fully understand in the entire region. Thus, there is a pressing need to examine species diversity patterns using current popular methods. Further research on the factors influencing Sternorrhyncha species diversity patterns in China will help shed light on the general explanation for the patterns of insect species diversity.

This paper aims to (1) analyze the species richness and endemism patterns of Sternorrhyncha in China, and (2) investigate the effects of multiple environmental variables on two types of patterns.

## ﻿Material and methods

### ﻿Geographic distribution patterns

A total of 15,450 distribution records of Sternorrhyncha species were obtained from previous studies (Gao 2018; Du et al. 2020; Li et al. 2021) and the Global Biodiversity Information Facility (https://www.gbif.org/). Species richness and endemism patterns were mapped by calculating the species numbers and weight endemism values in 1° × 1° grids, respectively. The weighted endemism of a grid was calculated as the sum of the range-down-weighted species values after each species was down-weighted by the number of grids in which it was found (Linder 2001). A grid size of 1° × 1° was chosen because it had been frequently used in previous studies on insect diversity patterns in China (Zhao et al. 2020a, 2021). We eliminated grids with less than half of their area in this study.

### ﻿Environmental variables

Thirteen environmental variables were selected to identify key ecological factors that drive patterns of species richness and endemism and they were grouped into six categories as follows: 1) ambient energy: mean annual temperature (MAT) and annual potential evapotranspiration (PET); 2) water availability: mean annual precipitation (MAP) and annual actual evapotranspiration (AET); 3) climate seasonality: temperature annual range (TAR), temperature seasonality (TS), and precipitation seasonality (PS); 4) habitat heterogeneity: elevation range (ER, calculated by maximum elevation minus minimum elevation in 1° grid) and slope (SP); 5) productivity: normalized difference vegetation index (NDVI) and net primary productivity (NPP); and 6) historical climate stability: MAT change and MAP change; these two variables were defined as the absolute value of the difference between the current MAT/MAP and the Last Glacial Maximum MAT/MAP. Because past climate models were uncertain, MAT and MAP in the Last Glacial Maximum were calculated as the mean of the Model for Interdisciplinary Research on Climate Earth system (MIROC-ESM) and Community Climate System Model v.4 (CCSM4) models.

TAR, TS, PS, and MAT and MAP at present and Last Glacial Maximum were obtained from the WorldClim database (http://www.worldclim.org). The NDVI and NPP were obtained from the Resource and Environment Data Cloud Platform (http://www.resdc.cn/). The PET and AET were downloaded from the CGIAR-CSI database (http://www.cgiar-csi.org). Elevation data were obtained from CGIAR SRTM (http://srtm.csi.cgiar.org/). The spatial resolutions of NDVI and NPP were 1 km^2^ and that of MAT and MAP in the Last Glacial Maximum was 2.5 arc min (~5 km). The spatial resolutions of other environmental variables were 30 arc-seconds (~1 km). Environment variable values were obtained in a 1° grid by calculating the mean of all pixels within it using ArcGIS v.10.5 (ESRI, Redlands, CA, USA).

### ﻿Statistical analysis

The reliability of the relationship between species diversity and environmental variables is affected by sampling bias. As a result, assessing sampling bias is an unavoidable step in biogeography. Based on previous studies, a linear regression model was developed using the square-root-transformed number of records and the observed richness in a 1° × 1° grid (Zhao et al. 2020b, 2021). The degree of sampling bias is indicated by the ratio of the observed species richness to the predicted species richness obtained from the linear regression model.

Pearson correlation analysis was used to assess the relationships between species richness/endemism and each environmental variable. Spatial autocorrelation is frequently found in data on species richness, increasing the rate of a type I error in a significance test (Diniz-Filho et al. 2003). To solve this problem, Dutilleul’s method was applied to estimate the number of degrees of freedom in correlation significance using Spatial Analysis in Macroecology v.4.0 software (Dutilleul et al. 1993; Rangel et al. 2010). Generally, the lowest value of the Akaike’s information criterion (AIC) was considered the best model to assess the relationship between species diversity and environmental variables. However, AIC is sensitive to the presence of spatial autocorrelation and produces minimum adequate models that are unstable and overfitted (Diniz-Filho et al. 2008). Therefore, a model averaging approach based on Akaike weights was used to obtain relative roles of environmental variables, and they were performed in R v.3.6.1 using the MuMIn packages (Bartón 2018; R Core Team 2019). To reduce the collinearity between the environmental variables, three environmental variables with high variance inflation factors, including, AET, TS, and MAT change, were excluded in the model averaging process. The variance inflation factors of the remaining environmental variables were less than 15. All possible environment variable combinations were considered to build models, and the relative role of each environment variable was defined as the sum of the AIC weights for all models in which it appeared. Species richness and endemism were log10-transformed, and all variables were standardized (mean = 0 and standard deviation = 1).

## ﻿Results

**Figure 1.**
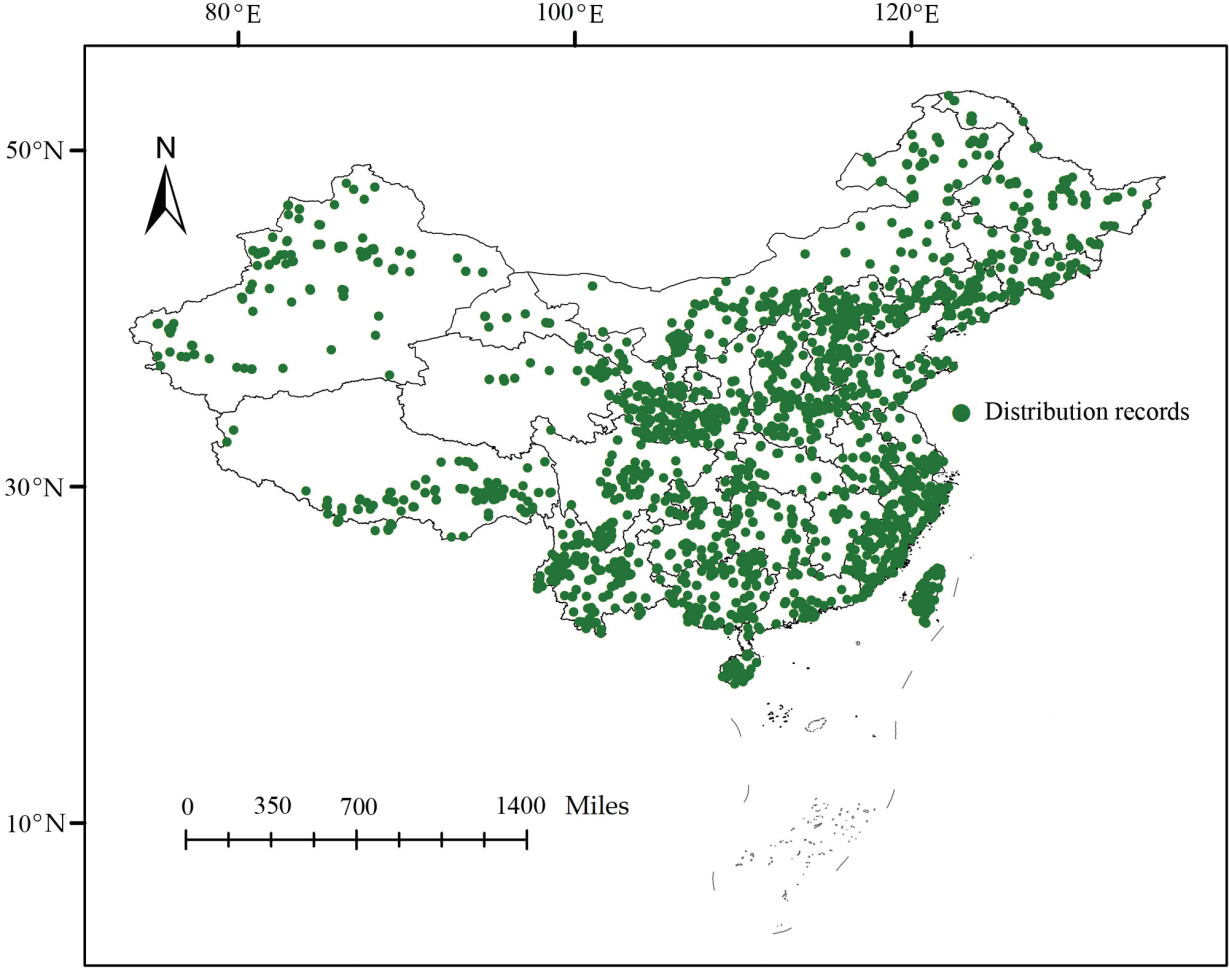
Distribution records of Sternorrhyncha in China.

**Figure 2.**
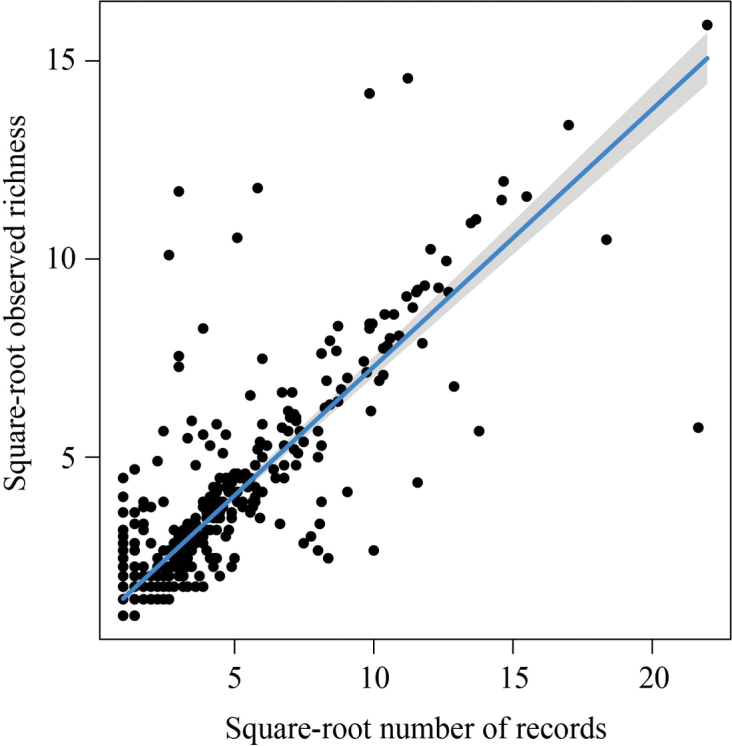
Linear regression (y = 0.649x + 0.781) for the square-root number of records and square-root observed richness.

**Figure 3.**
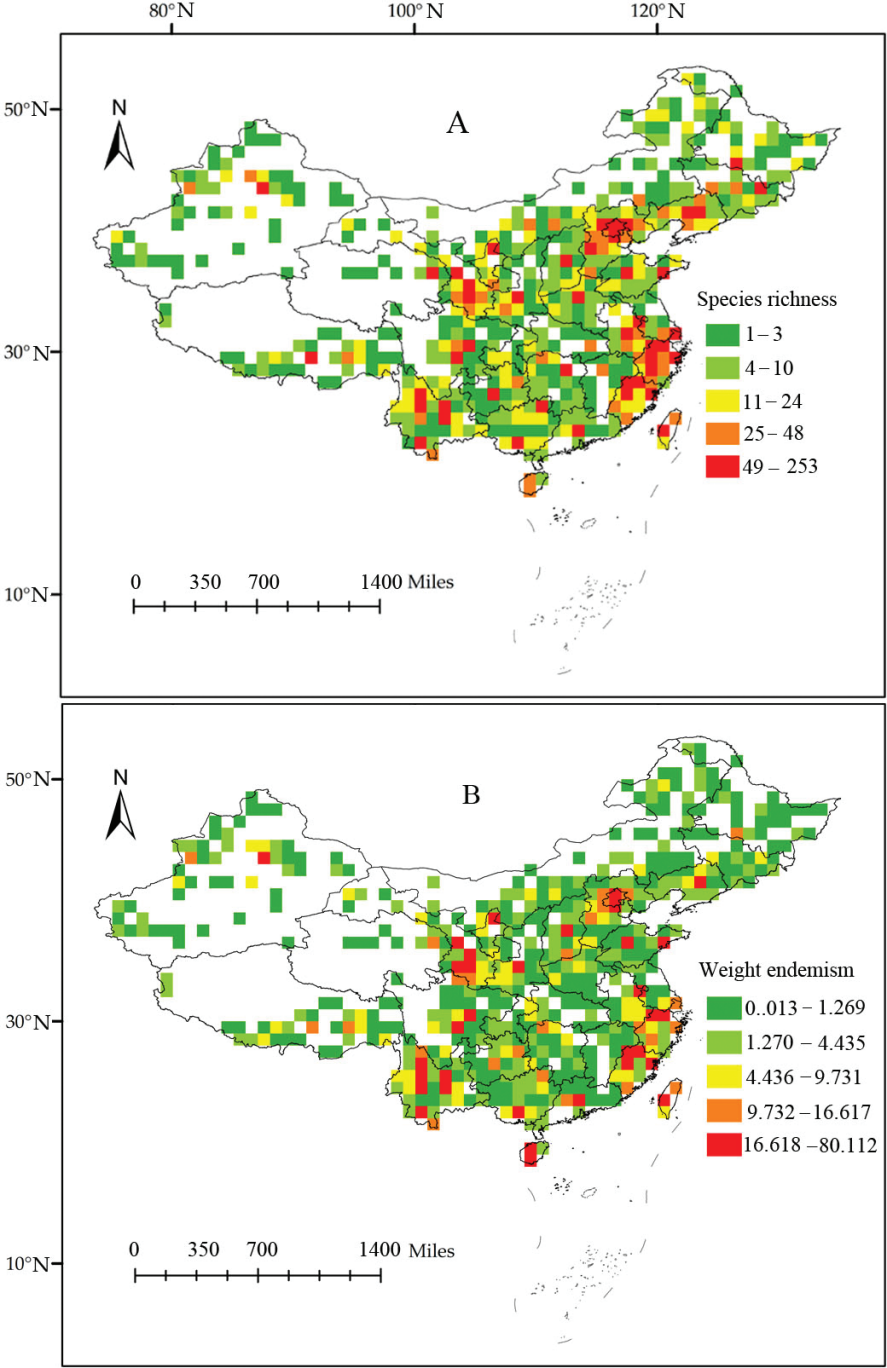
Species richness (**A**) and weight endemism (**B**) patterns of Sternorrhyncha in China.

All distribution records of Sternorrhyncha in China are shown in Fig. 1. The linear regression model revealed that the ratio of observed richness to the expected richness was greater than 69.93% for all grids (Fig. 2). Therefore, our results represent relatively good sampling. It is clear that the eastern and southeastern coastal regions of mainland China, as well as Taiwan Island, have high species richness (Fig. 3A). Endemism represented by the weight of endemism showed similar distribution patterns (Fig. 3B).

Correlation analysis revealed that species richness of Sternorrhyncha was strongly correlated with NDVI, ER and PET and moderately correlated with MAT, TAR and PS (Table 1). Other environmental variables were weakly correlated with species richness. Additionally, endemism was strongly correlated with ER and TAR and moderately correlated with NDVI, PET and TS (Table 1). The remaining environmental variables were weakly correlated with endemism (Table 1).

**Table 1.** Pearson correlations between species richness/endemism of Sternorrhyncha species and environmental variables.

Environmental variables		Species richness		Endemism	
		*r*	*p*	*r*	*p*
Ambient energy	MAT	0.520	0.016	0.462	0.03
	PET	0.761	0.195	0.661	0.019
Water availability	MAP	0.310	0.019	0.182	0.017
	AET	0.225	0.016	0.208	0.016
Climate seasonality	TAR	−0.536	0.010	−0.736	0.002
	TS	−0.114	0.020	−0.537	0.005
	PS	0.504	0.090	−0.416	0.008
Habitat heterogeneity	ER	0.783	0.039	0.842	0.033
	SP	0.224	0.030	0.303	0.045
Productivity	NDVI	0.846	0.003	0.633	0.002
	NPP	0.349	0.002	0.271	0.014
Historical climate stability	MAT change	0.193	0.050	0.168	0.034
	MAP change	0.382	0.040	0.182	0.017

**Figure 4.**
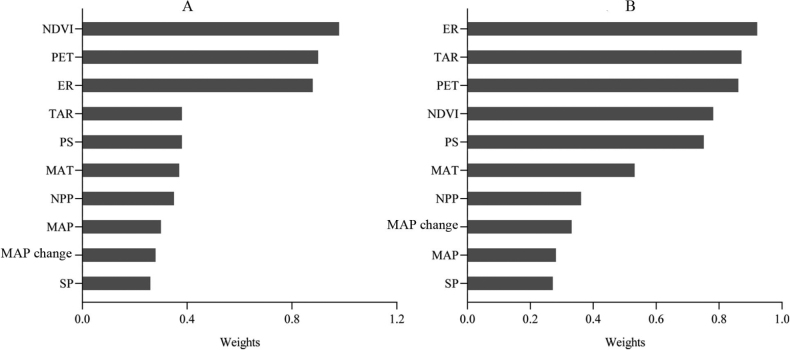
AIC weights of environmental variables in species richness (**A**) and endemism (**B**).

The model averaging approach revealed NDVI as the most important environment variable for species richness of Sternorrhyncha, followed by PET and ER (Fig. 4A). The role of other environmental variables was relatively low. The key environment variable for endemism was found to be inconsistent with that for species richness (Fig. 4B). The importance of ER ranked first, and TAR and PET ranked second and third, respectively; the remaining variables had a relatively low effect on endemism (Fig. 4B).

## ﻿Discussion

In this study, we found that some environmental variables had a substantial impact on the formation of species richness and endemism patterns of Sternorrhyncha species. The NDVI is widely regarded as a surrogate for plant productivity and has been shown to influence broad-scale patterns of species richness in plants and animals (Mittelbach et al. 2001; Kissling et al. 2007). Our findings revealed that NDVI is the most important environmental variable in shaping the patterns of Sternorrhyncha species richness (Fig. 4), as would be expected for a phytophagous insect. Consistent with our results, previous studies on the driving factor of species diversity patterns in other phytophagous insects, for example, planthoppers (Zhao et al. 2020b), birds (Qian et al. 2009b) and terrestrial mammals (Lin et al. 2009) in China have also found that plant productivity plays an important role. These results imply that plant productivity has become an indispensable factor that regulates patterns in species richness in China. The high species richness in a region with high plant productivity is often attributed to the increased capacity of accommodating population sizes (lower extinction rates in larger populations) in this region (Wright 1983; Evans and Gaston 2006). In this study, two surrogates of plant productivity (NPP and NDVI) were used, but NPP played a smaller role than NDVI (Fig. 4). This result differs from previous studies on other insect groups. For instance, Zhao et al. (2020b) found that NPP had a greater impact on planthopper species richness patterns in China than NDVI. Therefore, the contribution of these two environmental variables to species richness is related to taxa.

Considering the determinants of species richness patterns as the representative endemism patterns may lead to a biased understanding of the drivers of species richness patterns and hinder the development of conservation strategies (Jetz and Rahbek 2002). Therefore, the drivers of endemism patterns must be analyzed. The results of this study revealed that endemism patterns of Sternorrhyncha species are more related to topographic heterogeneity (Fig. 4). This is consistent with the viewpoint that species with a narrowing range worldwide are typically located in topographically complex regions (Jetz et al. 2004; López-Pujol et al. 2011). Several studies on insects and plants have shown that topographic heterogeneity plays a role in historical processes (i.e., as refuges), which accounts for the high correlations between topographic heterogeneity and endemism (Schuldt and Assmann 2009, 2011; López-Pujol et al. 2011). Therefore, combining our findings above, it is evident that there are differences in the mechanisms and variables that influence patterns of species richness and endemism. Additionally, this study supports that the range size of species cannot be ignored in understanding the geographic variation in species diversity. Studies have shown that endemism patterns are mostly driven by historical climate stability (Araújo et al. 2008; Rakotoarinivo et al. 2013; Zhao et al. 2020a). However, in this study, historical climate stability represented by MAP change did not dominate endemism patterns of Sternorrhyncha species, and the same was true for species richness patterns (Fig. 4).

PET, a surrogate of ambient energy, is the second and third important environmental variable for species richness and endemism patterns of Sternorrhyncha species (Fig. 4), respectively. Previous studies have reported that high ambient energy can promote species richness in a given region (Currie 1991; Qian 2013). The relative role of ambient energy and water availability in regulating species richness patterns has long been debated (Diniz-Filho et al. 2013; Xu et al. 2016; Liu et al. 2018). However, the role of these two environmental variables is closely related to the taxa and the geographical location of the region, according to a previous related study (Hawkins et al. 2003). In this study, we found that ambient energy was always more important than water availability (represented by MAP) for both species richness and endemism of Sternorrhyncha species (Fig. 4). This result may reflect that species range size does not dampen the relative importance of these two kinds of environmental variables. One of the main contributors to short-term climate stability is TAR, which in this study serves as the second important environmental variable for endemism patterns (Fig. 4). A short-term climate stability can promote the survival of a small range of species (Klopfer 1959; Klopfer and Macarthur 1960). The study on endemism patterns of Chinese Delphacidae and Gesneriaceae also demonstrated that short-term climate stability is a main driving factor (Liu et al. 2017; Zhao et al. 2020a).

## ﻿Conclusion

In summary, species richness and endemism patterns of Sternorrhyncha in China were investigated, and the relationship between environmental variables and these two kinds of species diversity patterns was further analyzed. The results showed that both species richness and endemism patterns were primarily concentrated in the eastern and southeastern coastal regions of mainland China, as well as Taiwan Island. Additionally, the predominant environmental variables for species richness and endemism patterns differed. Species richness patterns were most strongly correlated with NDVI, while endemism patterns were most strongly correlated with ER. The results highlight the importance of species range size in investigating the determinants of species diversity patterns. In this study, some important evolutionary/historical factors were not included (e.g., geological events and niche conservatism), because molecular data currently available is insufficient. Thus, once a complete phylogenetic tree using the molecular data was constructed in the future, the importance of evolutionary/historical factors can be determined.

## ﻿Additional information

### Author ORCIDs

Zhengxue Zhao https://orcid.org/0000-0003-1764-8690

### Data availability

All of the data that support the findings of this study are available in the main text.
